# Evolving Trends in Traumatic Hand Injury Diagnoses at a University Emergency Department—A 10-Year Analysis

**DOI:** 10.3390/jcm15093403

**Published:** 2026-04-29

**Authors:** Christian Missura, Esther Vögelin, Léna G. Dietrich

**Affiliations:** Department of Plastic and Hand Surgery, Inselspital, University Hospital Bern, University of Bern, 3010 Bern, Switzerland; christian.missura@outlook.com (C.M.); esther.voegelin@insel.ch (E.V.)

**Keywords:** hand injuries, emergency department, trauma epidemiology, injury complexity, triage, healthcare utilization, Switzerland, minor trauma, primary care access, outpatient care

## Abstract

**Background**: Hand trauma accounts for up to one-third of trauma-related emergency department (ED) visits and ranges from minor lacerations to complex multi-structural injuries. As healthcare systems, workflows, and patient behavior evolve, contemporary epidemiological data are crucial to guide triage, optimize resource allocation, and adapt patient care pathways. **Methods**: We performed a retrospective observational study of all hand-related ED consultations at a Swiss university hospital in 2013, 2016, 2019, and 2022. In total, 8644 cases were analyzed for demographics, diagnosis, anatomical localization, injury complexity (≥2 functional structures), seasonal distribution, and animal-related injuries. Temporal trends and demographic or clinical shifts were assessed using descriptive and inferential statistics. **Results**: Among 8644 cases, most patients were male (63.8%) with a median age of 38 years (IQR 26–55). Lacerations (32.2%) and blunt trauma (29.1%) were the most frequent diagnoses, primarily involving digits II–V. The proportion of complex injuries declined significantly from 40.0% in 2013 to 32.5% in 2022 (*p* < 0.001), while cat-bite injuries almost doubled from 1.3% in 2013 to 2.3% in 2022. Case volumes peaked in spring and early summer. **Conclusions**: Over the analyzed decade, hand trauma cases in the ED have shifted toward a rising proportion of minor, low-acuity conditions, possibly reflecting reduced primary care access and evolving referral patterns. These trends highlight the need for adaptive triage models, strengthened outpatient care, and structural responses to primary care shortages to ensure efficient resource use and maintain high-quality hand trauma management.

## 1. Introduction

The human hand plays a central role in cognition, communication, and daily function, reflecting its unique neuroanatomical and functional integration with the brain. Its complexity and versatility underline the importance of providing high-quality care when injuries or diseases compromise its structure or function [1].

Hand injuries represent a substantial share of trauma-related healthcare, accounting for up to 30% of all trauma-related emergency department (ED) visits [2,3]. These injuries can range from minor lacerations to complex multi-structural trauma, with potential for long-term functional impairment, psychological burden, and social limitations [4,5]. In addition to the direct impact on health, hand injuries impose a considerable economic burden due to prolonged work absence and associated productivity loss [6,7].

Despite their high prevalence and socio-economic impact, data on emergency consultations for hand trauma in Switzerland remains scarce. Most existing studies have been conducted in North America and Germany and have primarily focused on diagnostic distribution or demographic characteristics [8,9]. Only a few have explored changes in injury complexity or mechanisms of injury over time, and virtually none have addressed the evolving role of the emergency department in the management of such cases in the Swiss healthcare system.

This study addresses that gap by analyzing a structured dataset of hand-related emergency consultations collected in a major tertiary care center in Switzerland. By examining four distinct years (2013, 2016, 2019, and 2022), this study investigates trends in patient demographics, injury types, anatomical localization, complexity and seasonal variation. In doing so, it aims to better characterize current patterns of emergency care utilization for hand injuries and inform clinical decision-making, triage systems, and healthcare planning.

## 2. Materials and Methods

We conducted a retrospective analysis of hand emergency cases that presented to the emergency department. A hand injury is defined as damage to the structures of the hand or wrist, or damage to other structures that functionally impair or threaten to impair the hand or wrist. This inclusive definition extends to injuries of peripheral nerves that supply the hand, even if the damage occurs outside the immediate area of the hand. Similarly, injuries and diseases of the forearm that may affect structures serving the hand are included, given that they are often assessed and treated by hand surgeons. Excluded from this definition are pathologies that may involve the hand but are not primarily hand-related, such as cerebrovascular insults or widespread skin conditions.

The complexity of a hand injury is a key determinant of treatment strategy. Complex injuries are commonly defined as those involving damage to at least two distinct functional structures (e.g., skin, ligaments, tendons, bones, cartilage, nerves, arteries and veins or muscles), consistent with previously published definitions [10,11]. Complexity was solely assessed for hand injuries due to difficulty determining complexity of hand diseases with the mentioned definition.

### 2.1. Data Source

Data were extracted from structured Excel tables exported from the electronic medical record system of the University Emergency Department, Inselspital, University Hospital Bern, Switzerland. The extraction was performed by the intern data science center. All patients aged 16 years or older presenting with hand problems in 2013, 2016, 2019, and 2022 were included. Patients with multiple traumata were also included if one or more injuries involved the hand according to the inclusive definition of hand injury used in this study. Intervals of three years were chosen to gather data over a period of 10 years. The peak time of the COVID-19 pandemic (2020 and 2021) was avoided to show reliable long-term trends despite the effect of the pandemic. Variables comprised demographics (age, sex), diagnosis, anatomical localization, month of consultation, injury complexity, and animal-related injuries. All variables were pre-categorized and consistently structured across years, enabling direct comparisons.

### 2.2. Categorization Schema

**Figure A2:**
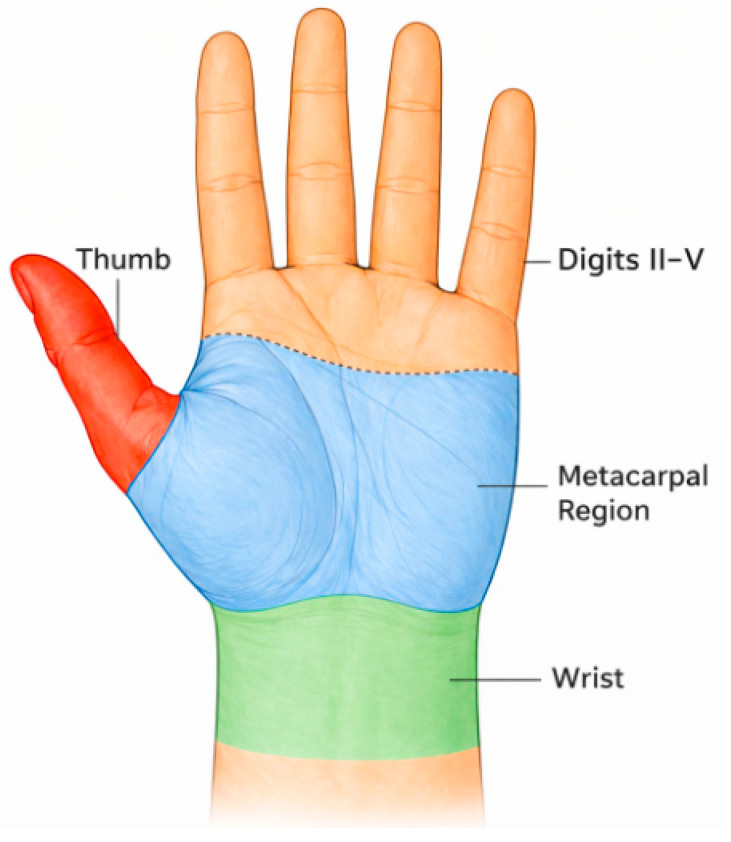
Anatomical regions of the hand used for classification. The hand was divided into four anatomical regions for analysis: digits II–V, thumb, the metacarpal region, and the wrist. The boundary between the digits and the metacarpal region corresponds to the metacarpophalangeal (MCP) crease.

A categorization schema was developed to analyze hand injuries and diseases. Unlike the extensive but fragmented ICD-10 classification, the schema provided a more concise structure tailored to this study. Categories were designed to include both injuries (e.g., fractures, lacerations) and diseases (e.g., infections, inflammations), anatomical localization (e.g., fingers, wrist), and injury complexity. Detailed mechanisms of injury were not consistently available in the dataset and were therefore not included as a separate analytical category. Cases were first categorized as either “Injury” or “Disease.” Each entry was then assigned a diagnosis, localization, and complexity. For example, a laceration with associated nerve damage would be classified as laceration, with additional notation of its complexity. For animal-bite injuries, the causal agent was noted. A depiction of the schema is available in Appendix A.1. In cases of multiple hand trauma, each case was classified according to the most complex injury, defined as the injury affecting the greatest number of distinct functional structures. Data categorization was performed by a postgraduate researcher and supervised by a clinician. In cases of missing data, classification was performed based on the available information; if a diagnosis could not be determined, the case was categorized as “other”. A schematic illustration of the anatomical regions used for classification is provided in Appendix A.2 Figure A2.

### 2.3. Statistical Analyses

Descriptive and inferential statistical methods were employed to analyze trends across the study period.

As age was not normally distributed, it is presented as median and interquartile range (IQR). Differences in age across the study years were assessed using the Kruskal–Wallis test. When significant differences were detected, post hoc pairwise comparisons were performed using Dunn’s test with false discovery rate (FDR) correction.

Yearly comparisons (2013, 2016, 2019, and 2022) were performed to identify temporal patterns in case volume and injury characteristics. For categorical variables, chi-square tests were used. Post hoc pairwise comparisons were conducted using Z-tests with FDR correction where appropriate.

For seasonal distribution, a chi-square goodness-of-fit test was used to analyze pooled data and Poisson regression was fitted for quantification.

For all analyses, a *p*-value of <0.05 was considered statistically significant. All statistical analyses were conducted using R (Version 4.3.2) [12], including descriptive summaries, cross-tabulations and correlation matrices.

### 2.4. Ethical Considerations

General informed consent was obtained prospectively from all patients as stated in the informed consent statement.

All data were extracted in a de-identified format from the electronic medical record system. No directly identifiable patient information was included in the dataset used for analysis. Data processing was conducted in accordance with institutional data protection standards and applicable national regulations.

## 3. Results

The results are presented across four main domains: demographic trends, diagnosis and anatomical localization, injury complexity and seasonal distribution. A summary of patient characteristics across the four study years is provided in Table 1. Values are presented as median (IQR) or percentages, as appropriate. *p*-values reflect overall comparisons across the four time points.

### 3.1. Demographic Trends

**Figure 1:**
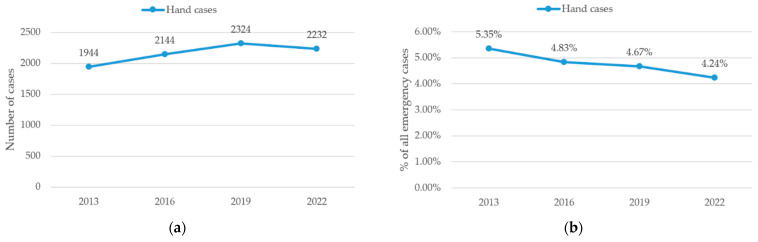
Number of hand cases. (**a**) Annual absolute hand case numbers. (**b**) Annual proportion of hand cases among all emergency department consultations. Data points represent discrete observations at the specified years; connecting lines are shown for visual guidance only and do not imply continuous trends between time points.

Total emergency consultations including all causes increased steadily from 36,329 in 2013 to 44,376 in 2016, 49,798 in 2019 and 52,703 in 2022. A total of 8644 hand cases were included across the four assessed years (2013, 2016, 2019, and 2022). The annual case numbers increased progressively from 1944 in 2013 to 2144 in 2016 and 2324 in 2019, followed by a slight decrease to 2232 in 2022, as presented in Figure 1a. Although the absolute number of hand cases increased over time, their relative proportion among all emergency department consultations declined, as the total number of emergency consultations increased more rapidly (Figure 1b).

The median age of patients remained stable across the study period, ranging from 37 years (IQR 25–53) in 2013 to 38 years (IQR 26–55) in 2022, with no significant differences observed (Kruskal–Wallis test, *p* = 0.17). No post hoc analysis was performed as no overall significant difference was detected.

**Figure 2:**
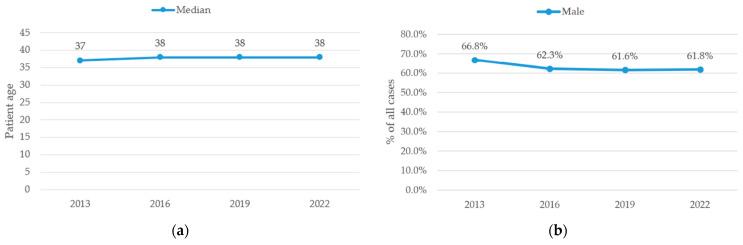
Demographic trends of hand cases. (**a**) Annual median patient age. (**b**) Annual proportion of male patients. Data points represent discrete observations at the specified years; connecting lines are shown for visual guidance only and do not imply continuous trends between time points.

Regarding sex distribution, male patients were consistently predominant across all years. However, their proportion decreased steadily from 66.8% in 2013 to 62.3% in 2016 and 61.6% in 2019, with a stabilization at 61.8% in 2022 (*p* < 0.001). Post hoc pairwise comparisons with FDR correction showed that 2013 differed significantly from 2016, 2019 and 2022 (all *p* < 0.001), while no significant differences were observed in between 2016, 2019 and 2022. This trend is illustrated in Figure 2b.

### 3.2. Diagnosis and Anatomical Localization

In this section, absolute case numbers per year are reported, with corresponding percentages of total hand emergency consultations provided in parentheses. For selected analyses, patients were stratified into four age groups: 16–29 years (*n* = 2864), 30–44 years (*n* = 2335), 45–59 years (*n* = 1886), and ≥60 years (*n* = 1559).

**Figure 3:**
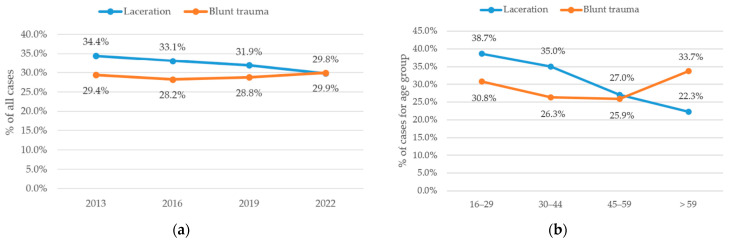
Distribution of the most common diagnoses of hand cases. (**a**) Annual percentage of diagnosis among all cases. (**b**) Annual percentage of diagnosis in different age groups. Data points represent discrete observations at the specified years; connecting lines are shown for visual guidance only and do not imply continuous trends between time points.

The most frequently documented diagnosis was laceration, representing 669 cases (34.4%) in 2013, 709 cases (33.1%) in 2016, peaking at 741 cases (31.9%) in 2019, and slightly declining to 665 cases (29.8%) in 2022. A significant decrease in relative proportions was observed (*p* < 0.001). Post hoc pairwise comparisons with FDR correction showed significant differences between 2013 and 2022 (*p* < 0.001) and between 2016 and 2022 (*p* = 0.004). All other comparisons were not statistically significant. Lacerations were followed by blunt trauma (including fractures, strains, and sprains), accounting for 571 cases (29.4%) in 2013, 605 cases (28.2%) in 2016, 669 cases (28.8%) in 2019, and 668 cases (29.9%) in 2022. No significant difference was observed (*p* = 0.59). Trends in diagnoses are shown in Figure 3a. Analysis across age groups revealed a significant predominance of lacerations among younger patients. In the 16–29-year age group, lacerations accounted for 38.7% of cases, decreasing progressively to 35.0% in the 30–44-year group, 27.0% in the 45–59-year group, and 22.3% in patients aged ≥ 60 years (*p* < 0.001). Post hoc pairwise comparisons including FDR correction showed that all pairwise comparisons between all groups were statistically significant (*p* < 0.001). This trend is illustrated in Figure 3b.

**Figure 4:**
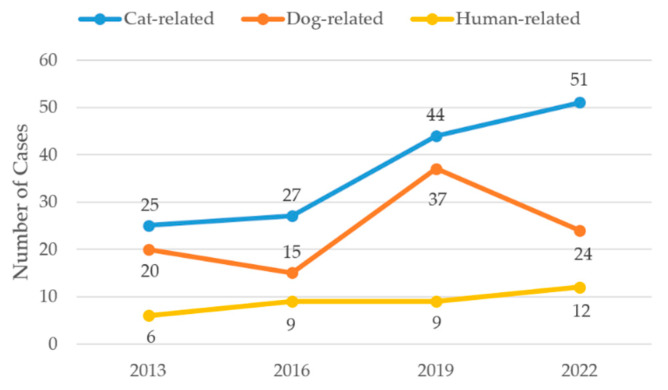
Annual case numbers of animal-related hand injuries. Data points represent discrete observations at the specified years; connecting lines are shown for visual guidance only and do not imply continuous trends between time points.

In terms of animal-related injuries, cat bites were predominant and increased over time. The number of cat-bite injuries was 25 cases (1.3%) in 2013 and 27 cases (1.3%) in 2016, rising to 44 cases (1.9%) in 2019 and 51 cases (2.3%) in 2022. Despite the relatively small case numbers, this trend showed to be globally statistically significant (*p* = 0.013), while post hoc pairwise comparisons including FDR correction revealed no statistically significant differences between individual years. Dog-related injuries showed greater variability, with 20 cases (1.0%) in 2013, 15 cases (0.7%) in 2016, 37 cases (1.7%) in 2019, and 24 cases (1.1%) in 2022, without a statistically significant trend (*p* = 0.1). Human-related injuries increased slightly over time, although they accounted for only a small proportion of cases, with 6 cases (0.3%) in 2013, 9 cases (0.4%) in 2016 and 2019, and 12 cases (0.5%) in 2022. This trend was not statistically significant (*p* = 0.36). Trends in animal-related injuries are shown in Figure 4.

**Figure 5:**
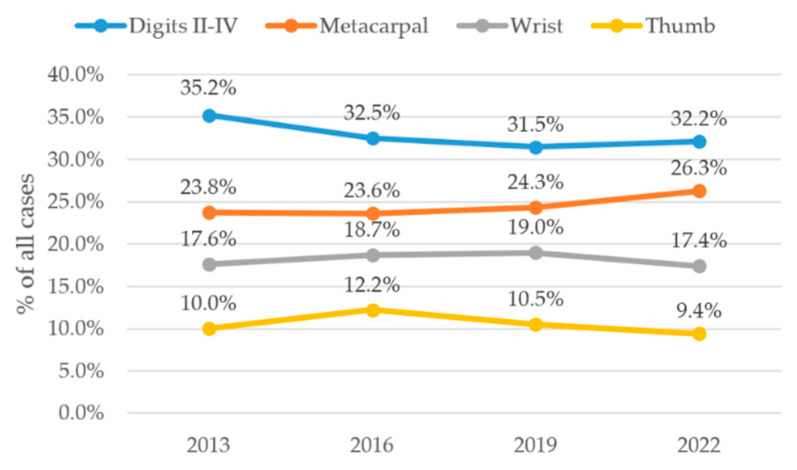
Annual distribution of the four most common anatomical localizations of hand cases. Data points represent discrete observations at the specified years; connecting lines are shown for visual guidance only and do not imply continuous trends between time points.

The most frequently affected anatomical localization were digits II–V in all analyzed years, with case counts ranging from 685 cases (35.2%) in 2013, 697 cases (32.5%) in 2016, 731 cases (31.5%) in 2019 and 718 cases (32.2%) in 2022. No statistically significant trend was observed over the four time points (*p* = 0.11). The second most common localization was the metacarpal region. A slight, steady increase of 462 cases (23.8%) in 2013, 506 (23.6%) in 2016, 565 (24.3%) in 2019 and 587 cases (26.3%) in 2022 showed to be statistically significant (*p* = 0.015). Yet, post hoc pairwise comparisons including FDR correction revealed no statistically significant differences between analyzed years. These cases were closely followed by affection of the wrist starting with 342 encounters (17.6%) in 2013, 401 (18.7%) in 2016, peaking at 441 (19.0%) in 2019 to then decline to 388 (17.4%) in 2022 (*p* = 0.27). Cases affecting the thumb showed case numbers of 195 in 2013 (10.0%), 262 in 2016 (12.2%), 244 (10.5%) and 210 (9.4%) (*p* = 0.04). Post hoc pairwise comparisons including FDR correction revealed no statistically significant differences between analyzed years. Trends of these four affected anatomical localizations are depicted in Figure 5.

### 3.3. Complexity of Injuries

**Figure 6:**
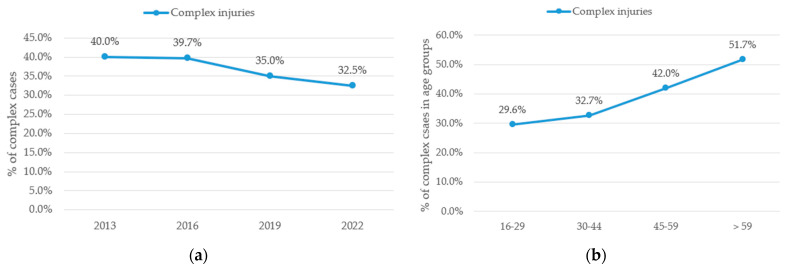
Complex injuries. (**a**) Annual percentage of complex injuries among all injuries. (**b**) Annual percentage of complex injuries in different age groups. Data points represent discrete observations at the specified years; connecting lines are shown for visual guidance only and do not imply continuous trends between time points.

The proportion of cases classified as complex injuries involving two or more distinct functional structures showed a consistent decline over the observed time period. In 2013, 645 cases (40.0%) were considered complex, a rate that remained nearly identical in 2016 with 702 cases (39.7%). However, the proportion decreased to 660 (35.0%) in 2019 and further to 586 (32.5%) by 2022, as demonstrated in Figure 6. The decreasing trend in complex injuries showed high statistical significance (*p* < 0.001). Post hoc analysis using pairwise comparisons with FDR correction revealed significant differences between 2013/2019 (*p* = 0.0048), 2013/2022 (*p* < 0.001), 2016/2019 (*p* = 0.0066) and 2016/2022 (*p* < 0.001). No significant differences were observed between 2013/2016 and 2019/2022. Notably, while the absolute number of complex injuries remained rather stable, the relative share of such cases in relation to the total case load gradually diminished as total case numbers increased.

Analysis for complexity of injuries among the different age groups showed strong correlation of age and complex injuries with rates of 29.6% for the youngest patients, 32.7% for patients aged 30–44 years, 42.0% for patients aged 45–59 years and 51.7% for patients aged > 60 years (*p* < 0.0001). Post hoc analysis using pairwise comparisons with FDR correction revealed significant differences (*p* < 0.0001) between all age groups except for 16–29 years vs. 30–44 years.

### 3.4. Seasonal Pattern

**Figure 7:**
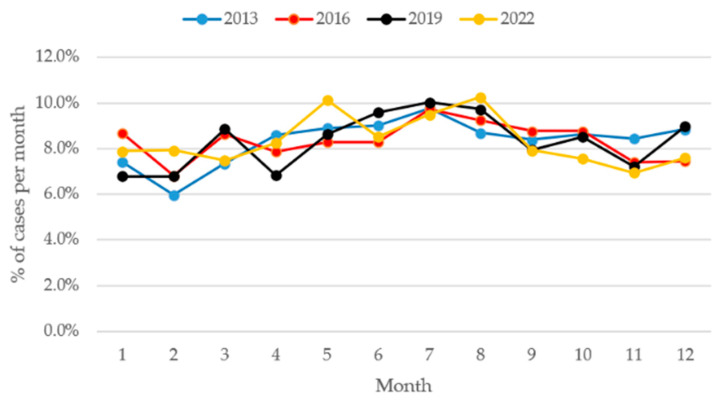
Percentage of hand cases by month. Data points represent discrete observations at the specified years; connecting lines are shown for visual guidance only and do not imply continuous trends between time points.

Monthly case distribution revealed distinct seasonal patterns across the four observed years. While some fluctuations were year-specific, several consistent trends emerged. Data of the seasonal distribution over these four years is shown in Figure 7. In all years, the number of hand cases tended to increase during spring and early summer months, particularly between April and July. Notably, May, July and August consistently represented months with high case numbers:

- In 2019, July had the highest monthly total of 233 cases, corresponding to 10% of all cases in 2019.
- In 2022, May and August reached peak values of 226 and 229 cases, respectively, accounting for 10.1% and 10.3% of total cases in 2022.

Conversely, February showed consistently lower case numbers across the years, with the lowest monthly totals observed with 116 cases (6.0% of annual cases) in 2013 and 146 cases (6.8%) in 2016.

When pooling data across all years, a highly significant deviation in monthly distribution was observed (*p* < 0.001). A Poisson regression model including month as a categorical variable showed a significant effect of month on case numbers (*p* < 0.001) with a peak in July (RR 1.27, 95% CI 1.15–1.40) compared to January) and a low in February (RR 0.90, CI 0.81–1.01) compared to January).

## 4. Discussion

Hand injuries are amongst the most prevalent and impactful conditions presenting to emergency departments worldwide, accounting for up to 30% of trauma-related visits [2,3]. They range from minor lacerations to complex injuries involving multiple anatomical structures and represent a significant public health, clinical, and economic burden [4,5,6,7]. By providing longitudinal data over nearly a decade from a Swiss tertiary care center focusing on diagnostic trends, injury complexity, and demographic shifts, this study complements existing epidemiological evidence from North America and Germany. It thereby contributes to a more comprehensive understanding of global trends in hand trauma and its treatment.

### 4.1. General Trend Analysis and Interpretation

Over the observed time period from 2013 to 2022, several notable trends emerged in the epidemiology of hand cases.

Case volume increased steadily from 1944 cases in 2013 to a peak of 2324 in 2019, before slightly declining to 2232 in 2022 as shown in Figure 1a. This observation is in line with previous European data, which also demonstrated a continuous increase in hand trauma cases and a gradual rise in patient age over time [13]. This pattern suggests a rising incidence of hand cases up to 2019, potentially reflecting growing clinical capacity, increasing population activity levels, or improved documentation. The modest decline in 2022 may be related to external factors such as healthcare access fluctuations during or after the COVID-19 pandemic.

The median age of patients remained stable over the study period, with no statistically significant differences between years (Figure 2a).

A steady, significant decline in the proportion of male patients was observed: from 66.8% in 2013 to 61.8% in 2022 (Figure 2b). This trend may suggest either increased injury rates among females or relatively decreasing exposure among males, possibly due to shifts in workplace structures or domestic injury patterns.

Injury patterns remained rather stable in terms of diagnosis and localization as visible in Figure 3 and Figure 5. Lacerations were the most frequent diagnosis and declined significantly, followed by blunt trauma which remained stable. Lacerations were strongly predominant in younger patients as shown in Figure 3b.

Likewise, injuries to the digits II–V were the most common anatomical localization throughout the observation period followed by metacarpal region and wrist thereafter. Despite a mild increase in metacarpal injuries and modest decrease in affection of the thumb, affection of the anatomical regions remained remarkably stable. These patterns confirm the substantial and ongoing prevalence of hand trauma, even in well-developed healthcare systems and underline the functional and exposure-related vulnerability of this anatomical region during occupational and domestic activities. Our findings align with previous studies showing that the fingers, particularly digits II–V, are the most commonly affected anatomical region, followed by the metacarpals and wrist [2,8].

One noteworthy trend was the increasing frequency of cat-bite injuries, which nearly doubled from 1.3% of cases in 2013 to 2.3% in 2022 as depicted in Figure 4. This may reflect rising pet ownership, increased awareness of zoonotic injuries, or improved recording practices in clinical documentation. During the COVID-19 pandemic, the surge in pet ownership may have led to increased exposure to domestic animals. This could therefore have directly translated into a higher frequency of such consultations [14]. Although relatively uncommon in absolute numbers, these injuries carry a high risk of infection or toxic reactions of the affected tissue and may require surgical intervention. This trend also underlines the need for awareness campaigns and early antibiotic strategies, especially for immunocompromised individuals or injuries involving deep puncture wounds [15,16].

Overall, the data demonstrates a gradual evolution in patient demographics and a stable injury pattern, with slight fluctuations likely attributable to social, occupational, or environmental changes over time. This shift is supported by previous studies demonstrating a significant increase in less severe injuries such as superficial lacerations, sprains, and minor fractures over time [13].

### 4.2. Trends in Injury Complexity

One of the most significant findings was a statistically significant decline in the proportion of complex injuries from 2013 to 2022 as shown in Figure 6a. In 2013, 40% of cases were classified as complex, compared to only 32.5% in 2022. Complex injuries appeared to be more prevalent in older patients (>59 years), as shown in Figure 6b. This is consistent with previous findings indicating that elderly patients are more likely to sustain more severe and complex injuries requiring advanced treatment strategies [13] and could represent higher vulnerability of this specific patient group. Similar declining trends of complex injuries have been reported in other European cohorts [13]. These findings are consistent with previous epidemiological studies, although differences may reflect variations in healthcare systems, population behavior, and classification methods.

This downward trend may reflect either a true epidemiological shift, such as earlier patient presentation, improved preventive strategies, or changes in injury mechanisms, or an evolution in documentation criteria or classification practices. Improved preventive measures such as stricter workplace safety regulations, advances in protective equipment, and the increasing use of safer machinery and tools in occupational settings could have contributed to a genuine reduction in the incidence of multi-structural hand trauma [17]. However, it must also be noted that complexity classification was based on a binary coding system, which lacks standardized criteria and may be influenced by inter-observer variability [10,11].

The decline in complexity also raises questions about evolving referral practices and triage thresholds. As hand surgery becomes more subspecialized, general emergency physicians may be more cautious in labeling injuries as “complex,” potentially contributing to this trend.

Another explanation for this trend could be that patients with less complex injuries increasingly present to emergency departments, potentially reflecting reduced access to primary care or specialist outpatient services [18]. This shift may reflect broader structural changes in healthcare access, including reduced availability of primary care services and increasing reliance on emergency departments for low-acuity conditions.

Our findings suggest a potential decreasing injury-severity or case selection over time, although prospective validation and standardized definitions of complexity would be needed to substantiate this observation.

### 4.3. Seasonal Pattern

As shown in Figure 7, our data suggests a clear decline in cases during winter months. This may reflect decreased outdoor or manual activity levels, reduced sports participation, and lower occupational exposure due to seasonal work patterns [19,20].

These seasonal variations underscore the potential influence of environmental, occupational, and behavioral factors on hand trauma incidence. They may also have implications for staffing, resource allocation, and preventive public health measures during high-incidence months [21].

### 4.4. Clinical and Epidemiological Insights

Despite being mostly non-life-threatening, hand injuries pose diagnostic and therapeutic challenges. While minor injuries may lead to temporary discomfort, more severe cases can result in long-term disability, functional loss, and psychological distress [5,7]. Mechanisms of injury are diverse and often linked to occupational, recreational, or domestic activities, requiring targeted preventive strategies [22].

Interestingly, although the absolute number of hand cases remained relatively stable (Figure 1a), their proportion relative to total emergency consultations at the university hospital in Switzerland considered in this study slightly declined over time (Figure 1b). This could indicate shifting healthcare utilization patterns, increased workplace safety, or changes in emergency department triage and coding.

### 4.5. Psychological and Functional Consequences

Hand injuries often lead to chronic limitations in daily activities, occupational performance, and mental health. Richards (2011) has emphasized the prevalence of psychological distress, such as anxiety and depression, among patients undergoing emergent hand surgery [7]. These sequelae persist beyond physical recovery and are often underestimated in trauma care.

Cook et al. (2017) found that, despite 40% of patients with mangled hand injuries being at risk for post-traumatic stress disorder (PTSD), only 22% were referred for psychiatric evaluation, leading to the implementation of a universal PTSD screening protocol in their clinic [23]. Moreover, Giladi et al. (2014) highlighted that standard outcome measures after traumatic finger amputations often fail to capture the psychological burden [24].

The persistent psychological sequelae of hand trauma highlight the importance of integrating mental health services into routine follow-up care, ensuring that recovery encompasses both physical function and psychosocial well-being.

### 4.6. Economic Burden

The economic implications of hand injuries are substantial. De Putter et al. estimated annual healthcare and productivity costs related to hand and wrist trauma to exceed $740 million in the Netherlands alone, with indirect costs, particularly work absenteeism, being the major contributor [6]. In our cohort, the predominance of working-age patients reflects this burden, particularly among younger adults and men. These findings are consistent with studies showing that economically active populations are disproportionately more affected, leading to significant productivity loss [25]. In Switzerland, the economic impact of hand injuries is likely amplified by the high labor costs and the predominance of full-time employment among working-age adults. Even short periods of work absenteeism may therefore translate into disproportionately high productivity losses compared to many other healthcare systems [26].

Strategies to reduce recovery time and facilitate return to work, such as early rehabilitation, occupational therapy, and employer accommodations, could substantially mitigate these costs. Public health authorities and insurance providers should consider the high return on investment of such measures in this injury category.

### 4.7. Prevention and Management Strategies

Preventing hand injuries requires a multifaceted approach. Targeted strategies include enforcing workplace safety regulations, promoting the use of protective equipment, and running educational campaigns for both industrial and domestic settings [22]. At the healthcare level, specialized hand trauma units, as recommended by Gordon et al., may improve outcomes through rapid diagnosis and coordinated care [9].

A notable contextual factor in Switzerland is the growing shortage of general practitioners, particularly in rural and semi-urban regions [18,27]. This structural deficit in primary care has led to an increasing reliance on emergency departments for conditions that would traditionally be managed in an outpatient setting. The observed increase in low-acuity hand cases may support the implementation of alternative care pathways, such as referral to general practitioners or dedicated fast-track emergency units for minor conditions. In addition, the identified seasonal peaks in spring and summer may help guide staffing strategies and resource allocation within emergency departments. This targeted allocation of resources could improve efficiency while ensuring that complex cases continue to receive specialized care. Our data reflects this shift, as a considerable proportion of hand trauma cases, particularly less complex injuries, presented directly to the emergency department. While this ensures rapid access to care, it also contributes to ED overcrowding and may divert resources from more urgent cases. Addressing this challenge will require health system reforms that strengthen primary care infrastructure and promote appropriate triage systems that can differentiate between low-acuity presentations and complex trauma. This could ensure that specialized surgical resources are reserved for patients with the greatest need.

### 4.8. Limitations

This study has several limitations. First, it represents a single-center analysis from a university emergency department, which may limit generalizability to other healthcare settings. Second, its retrospective design relies on the accuracy and completeness of electronic records, introducing potential misclassification or documentation bias, particularly regarding injury complexity. Third, only four time points were included, rather than continuous yearly data, which may not fully capture short-term fluctuations or external influences such as the COVID-19 pandemic. More recent data beyond 2022 were not included, which may limit the ability to capture the most current trends, particularly in light of recent changes in lifestyle and mobility patterns. Finally, referral patterns and changes in primary care access were not directly measured; so, the observed increase in non-complex cases can only be inferred. Despite these limitations, the large case number and standardized categorization across a decade provide robust insights into evolving patterns of hand trauma care.

While limited by its single-center design, the large sample size and standardized categorization strengthen the validity of the observed trends.

### 4.9. Implications for Future Research

The evolving patterns of hand injuries highlight the need for prospective multicenter studies that integrate clinical, psychological, and economic outcomes. Such research is essential not only to optimize rehabilitation protocols and evaluate cost effectiveness, but also to better understand the rising incidence of non-complex cases that place a disproportionate burden on university emergency departments, despite treatment options being available in peripheral settings. By addressing long-term outcomes, tailored rehabilitation, and healthcare access, evidence-based reforms in hand trauma care can be advanced.

## 5. Conclusions

Hand injuries remain a major clinical and socioeconomic burden. Our findings reveal a rising proportion of non-complex cases that increasingly strain university emergency departments despite the availability of peripheral treatment options. Prospective multicenter studies integrating functional, psychological, and economic outcomes are needed to clarify underlying causes, evaluate tailored rehabilitation protocols, and assess cost effectiveness. Such evidence will guide reforms in triage, healthcare access, and resource allocation to improve outcomes and reduce societal costs.

## Figures and Tables

**Table 1:** Patient characteristics across the four study years.

Variable	2013 (*n* = 1944)	2016 (*n* = 2144)	2019 (*n* = 2324)	2022 (*n* = 2232)	*p*-Value
Age (median [IQR])	37 (25–53)	38 (26–53)	38 (26–55)	38 (26–55)	0.17
Male (%)	66.8%	62.3%	61.6%	61.8%	<0.001
Lacerations (%)	34.4%	33.1%	31.9%	29.8%	<0.001
Blunt trauma (%)	29.4%	28.2%	28.8%	29.9%	0.59
Cat-bite injuries (%)	1.3%	1.3%	1.9%	2.3%	0.013
Complex injuries (%)	40.0%	39.7%	35.0%	32.5%	<0.001

**Figure A1:**
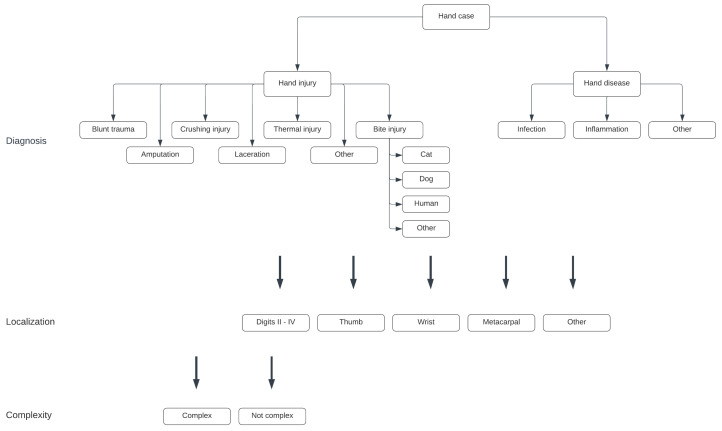
Categorization schema. The schema illustrates the structured approach used to classify hand-related cases into predefined categories, including injury versus disease, diagnostic category, anatomical localization, and injury complexity. Each case was assigned to a single primary diagnostic category, while complexity was defined as involvement of two or more distinct functional structures.

## Data Availability

The data underlying this study are not publicly available due to institutional and privacy restrictions.

## Abbreviations

The following abbreviations are used in this manuscript:

ED	Emergency department
